# Landslide Susceptibility Mapping by Fusing Convolutional Neural Networks and Vision Transformer

**DOI:** 10.3390/s23010088

**Published:** 2022-12-22

**Authors:** Shuai Bao, Jiping Liu, Liang Wang, Milan Konečný, Xianghong Che, Shenghua Xu, Pengpeng Li

**Affiliations:** 1 School of Geomatics, Liaoning Technical University, Fuxin 123000, China; 2 Chinese Academy of Surveying and Mapping, Beijing 100036, China; 3 Laboratory on Geoinformatics and Cartography, Department of Geography, Masaryk University, 61137 Brno, Czech Republic; 4 Faculty of Geosciences and Environmental Engineering, Southwest Jiaotong University, Chengdu 610031, China

**Keywords:** landslide, attention, convolution, deep learning

## Abstract

Landslide susceptibility mapping (LSM) is an important decision basis for regional landslide hazard risk management, territorial spatial planning and landslide decision making. The current convolutional neural network (CNN)-based landslide susceptibility mapping models do not adequately take into account the spatial nature of texture features, and vision transformer (ViT)-based LSM models have high requirements for the amount of training data. In this study, we overcome the shortcomings of CNN and ViT by fusing these two deep learning models (bottleneck transformer network (BoTNet) and convolutional vision transformer network (ConViT)), and the fused model was used to predict the probability of landslide occurrence. First, we integrated historical landslide data and landslide evaluation factors and analysed whether there was covariance in the landslide evaluation factors. Then, the testing accuracy and generalisation ability of the CNN, ViT, BoTNet and ConViT models were compared and analysed. Finally, four landslide susceptibility mapping models were used to predict the probability of landslide occurrence in Pingwu County, Sichuan Province, China. Among them, BoTNet and ConViT had the highest accuracy, both at 87.78%, an improvement of 1.11% compared to a single model, while ConViT had the highest F1-socre at 87.64%, an improvement of 1.28% compared to a single model. The results indicate that the fusion model of CNN and ViT has better LSM performance than the single model. Meanwhile, the evaluation results of this study can be used as one of the basic tools for landslide hazard risk quantification and disaster prevention in Pingwu County.

## 1. Introduction

A landslide is a natural phenomenon in which soil or rock on a slope slides downhill, either as a whole or in a scattered manner, under the influence of gravity [1]. Landslides mainly occur in areas with large undulations, little vegetation cover, frequent construction, aftershocks and heavy rainfall and broken rocks [2]. The main hazards of landslides are the destruction of farmland and buildings, injury to people and animals and destruction of forests. They are also economically harmful in water and electricity projects, roads, railways, river transport and marine projects. In addition, landslides can also cause secondary disasters that endanger human life and property [3,4].

Landslide susceptibility mapping (LSM) is a comprehensive analysis of various geological and environmental factors, historical landslide data, physical patterns of landslides and other elements in the study area to predict the spatial and temporal distribution and probability of landslide hazards [5]. The prediction results can provide an important decision basis for regional landslide hazard risk management, territorial spatial planning and landslide decision making [6]. The LSM methods are mainly divided into qualitative and quantitative evaluations [7]. Qualitative evaluation mainly includes landslide cataloguing and knowledge-driven, which has the advantage of being a simple method, more operable and more adequate in expressing the intrinsic mechanism of landslide hazards but has the disadvantage of relying more on a priori knowledge and weaker objectivity and is suitable for a small study area [8]. A quantitative evaluation mainly relies on mathematical knowledge to establish probabilistic statistical models, specifically including statistical methods [9,10] and machine learning (ML) methods [11], as well as deep learning (DL) methods [12] and other artificial intelligence methods [13]. Quantitative evaluation relies less on a priori knowledge and is more objective than qualitative evaluation, making it suitable for application to a large range of regions. Quantitative evaluation is more capable of handling high-dimensional data and massive amounts of data, and the models have a strong generalisation capability.

With the rise of artificial intelligence methods, ML and DL are widely used in the fields of geohazard prediction and geohazard identification. Decision trees [14], random forests [15], support vector machines [16,17], logistic regressions [18] and other methods have achieved good results in LSM. LSM methods based on ML mainly perform direct classification for the input data, without extracting high-dimensional features from the input data to improve classification accuracy and generalisation. At the same time, ML is prone to produce locally optimal solutions due to overfitting.

DL can effectively overcome the shortcomings of traditional ML models and efficiently extract deep and intrinsic features of data through multi-layer neural networks. Convolutional neural networks (CNN) [19,20], recurrent neural networks (RNN) [21], the combination of CNN and RNN [22], and the combination of CNN and ML have also made good progress in the field of LSM [23,24,25]. CNN use a fixed size convolutional kernel and repeatedly ‘convolve’ the information around the kernel layer by layer, gradually expanding the field of perception [26,27]. The purpose of feature extraction of the input data is thus achieved. However, the local feature extraction method of CNN does not retain enough spatial information, and thus cannot improve the classification accuracy.

Vision Transformer (ViT) combines computer vision and natural language processing domain knowledge by chunking the original image, spreading it into a sequence, feeding it into the encoder part of the original transformer model, and finally accessing a fully connected layer classifies the images [28]. ViT has more similarity between the shallow and deep representations, and the global representation is obtained from the shallow layer, which also retains more spatial information [29]. However, ViT’s Self-Attention induction bias is weaker than that of CNN, so ViT needs more data to fit the network model.

The limited perceptual field of CNNs makes it difficult to capture global information, while the Transformer can capture long-range dependencies. Therefore, since the emergence of ViT, there have been many attempts to combine CNN and Transformer, so that the network structure can inherit the advantages of both CNN and Transformer and retain the maximum amount of global and local features. The combination of CNN and ViT to complement each other’s strengths is one of the current hot spots in the field of LSM, and indeed in DL. In this study, we use CNN and ViT for LSM. Based on this, we use the Residual Neural Network (ResNet) model in CNN as the basis and fuse the Multi-Head Self-Attention (MHSA) module in ViT to generate the bottleneck transformer network (BoTNet). Meanwhile, ViT was used as the basis for fusing soft convolution-induced biases to stimulate the network to perform convolutional operations to generate convolutional vision transformer network (ConViT) models for application to LSM. This is the first application of the fused model of CNN and ViT to the LSM domain. At the same time, we analyse what advantages each of CNN and ViT has for LSM tasks. The possibility of deep fusion between the CNN and ViT models is also analysed.

The main objectives of this study are:

(1) To explore the accuracy differences and generalisation capabilities of CNN and ViT on the field of LSM.
(2) To investigate the prediction accuracy and applicability of the models after the fusion of CNN and ViT with each other.
(3) The advantages and disadvantages of CNN and ViT in LSM are compared, and the feasibility of deep fusion between CNN and ViT is analysed.

## 2. Materials and Methods

### 2.1. Study Area

**Figure 1:**
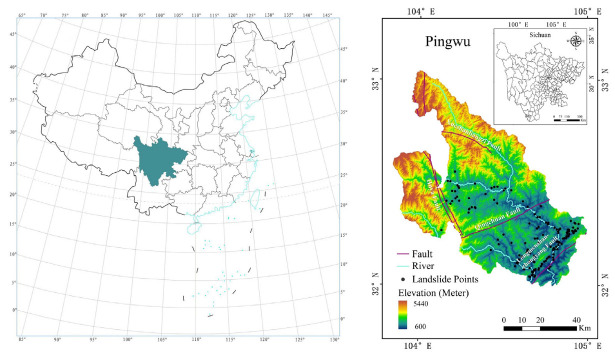
Study area and landslide distribution.

Pingwu County (103°50′ E–104°58′ E, 31°59′ N–33°02′ N) is located in the northern part of Mianyang City, Sichuan Province, China, as shown in Figure 1, with an area of 5974 km^2^. It is located on the eastern edge of the transition from the Qinghai-Tibet Plateau to the Sichuan Basin, in the upper reaches of the Ful River, a secondary tributary of the Yangtze River. The geotectonic position of Pingwu County is at the western edge of the Yangzi Quasi-Terrestrial Platform in the Longmenshan Fold Fault Zone and the Kunlun-Qinling Trough Fold Zone in the West Qinling-Motianling East-West Tectonic Zone and the Songpan-Ganzi Trough Fold Zone, which is an earthquake-prone area [30]. The strata of the Mesozoic Jurassic and all previous geological periods are basically complete.

The topography of the county is high in the northwest and low in the southeast, with the highest point at an altitude of 5440 m and the lowest at 600 m, with a height difference of 4840 m, and the area of mountains above 1000 m above sea level accounts for 94.33% of the area. The average annual temperature in Pingwu County is 14.7 °C, the average annual relative humidity is 71% and the average annual sunshine is 1376 h. It rains on average 15 days a month from May to October. June to September is the flood season in Pingwu, with average monthly rainfall greater than 100 mm. The terrain is very undulating, and the three-dimensional climate varies significantly, belonging to the northern subtropical mountain monsoon climate. Rainfall is concentrated and intense; the landform types are intricate and complex, with large slopes, broken rocks, severe weathering and low resistance to erosion; indiscriminate logging, rough farming, unreasonable land use and more serious soil erosion. The average annual soil erosion area is 2097 km^2^, accounting for 35.2% of the total area, and the total annual soil erosion is 258.5 million m^2^.

### 2.2. Data Sources

Landslide data for this study were obtained from the Resource and Environmental Science and Data Centre of the Chinese Academy of Sciences (https://www.resdc.cn/data.aspx?DATAID=290, accessed on 1 March 2022). There are 150 historical landslides in the study area, and the attribute information includes geographical location, groundwater type, tectonic site and seismic intensity. These landslide sites were corrected in combination with field surveys and remote sensing imagery.

In this study, landslide-related data were collected to compose a total of 11 landslide conditioning factors in 4 categories.

(1) Topographic factors: elevation, slope, aspect, plan curvature and profile curvature.
(2) Geological factors: distance to fault and lithology.
(3) Hydrological factors: distance to river and rainfall.
(4) Ecological factors: landcover and normalised difference vegetation index (NDVI).

Slope is the steepness of a surface unit and is usually called the quotient of the vertical height of the slope and the distance in the horizontal direction. Aspect is the direction in which the normal vector of the slope is projected on the horizontal plane. Plane curvature is the curvature of the surface unit along the direction of slope, and profile curvature is the curvature of the surface unit perpendicular to the direction of slope. Topographic data reflect the elevation, steepness, exposure to light and complexity of the terrain of the surface unit [31]. The shear strength of a slope varies with the lithological composition of the surface and the ease with which landslides can occur. We calculate the Euclidean distance from any location within the study area to the nearest fault and river, defined as distance to fault and distance to river, respectively [32]. The study area contains faults and the distance to the fault laterally reflects the probability of a landslide occurring. Rainfall disrupts the equilibrium of slopes, leading to sliding phenomena ranging from slow and persistent slope deformation to sudden and massive eruptions. Surface cover influences erosion, rainfall, water infiltration, slope loading and the structural characteristics of the ground, which increase the likelihood of slope instability or directly induce landslides by creating footing ratios, altering the shear resistance of sliding bodies and creating hard and soft surface structures. Normalised vegetation indices are obtained by measuring reflectance values in the near-infrared and infrared wavelengths and respond well to ecosystem structure. The reinforcement of the soil by vegetation is accomplished by the subsurface biomass, the higher the biomass in the subsurface part, the stronger the reinforcement [33].

**Figure 2:**
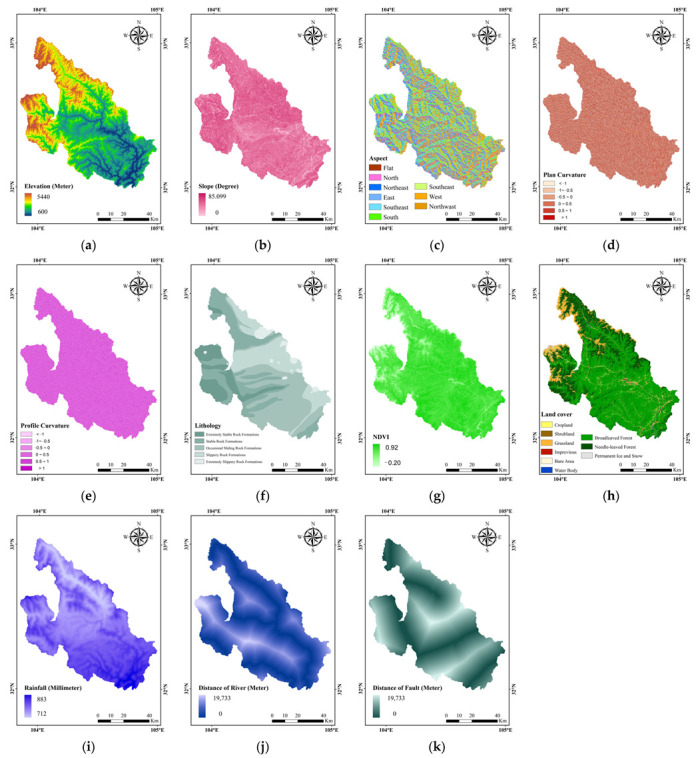
Landslide conditioning factors of LSM. (**a**) Elevation, (**b**) Slope, (**c**) Aspect, (**d**) Plan curvature, (**e**) Profile curvature, (**f**) Lithology, (**g**) NDVI, (**h**) Landcover, (**i**) Rainfall, (**j**) Distance to river and (**k**) Distance to fault.

Slope, aspect, plane curvature and profile curvature data are derived from elevation data. Topographic data from China Academy of Surveying and Mapping Sciences Beijing Four Dimensions Digital Technology Co. The landcover data is a global 30 m land cover classification data generated by combining the time series of Landsat images with high quality training data from the Global Spatial Temporal Spectra Library on the Google Earth Engine computing platform [34,35]. The NDVI data is based on the Google Earth Engine computing platform, using all Landsat5/7/8 remote sensing data for the whole year, obtaining all Landsat valid observations by de-clouding and de-shadowing, then extracting NDVI for each Landsat valid observation and combining linear interpolation and S-G smoothing methods to obtain the NDVI maximum for each image element in a year, forming the 30 m annual maximum NDVI dataset for China from 2000 to 2020 was obtained. The spatial resolution of the dataset is 30 m, and the temporal resolution is 1 year. The rainfall data were extracted from the 1991–2020 average rainfall in China with a spatial resolution of 30 m. The rainfall data were cross-validated with the ERA5 precipitation data from the Climate Reanalysis Information and the 30-year climate standard period 1991–2020 from the Hong Kong Observatory. Lithology, rainfall, landcover and NDVI data are from the Chinese Academy of Sciences. The source data were saved as integer raster data by magnifying the rainfall data by a factor of 10 to save storage space, and the NDVI data were saved with the value range transformed from [−0.2, 1] to [0, 255]. The data sources, data types, statistical information and spatial distribution of the landslide conditioning factors are shown in Table 1 and Figure 2.

### 2.3. Methods

#### 2.3.1. CNN

The CNN takes the raw data as the input to the algorithm, and through a series of operations such as convolution, pooling and nonlinear activation function mapping, the raw data is abstracted layer by layer into the final feature representation required for its own task and finally ends with a mapping of features to the task target. Although there are many variants of CNNs, they all have a very similar structure, consisting of input layers, convolutional layers, pooling layers, fully-connected layers and output layers.

The main problems of the network depth in deep learning are gradient disappearance and gradient explosion, which have been solved to some extent by the emergence of ResNet, whose initial goal is *H*(*x*), but as the number of layers increases, learning *H*(x) becomes increasingly difficult. As a result, the learning objective becomes *F(x) = H(x) − x*, where *F(x)* is the residual. This learning process is referred to as residual learning [36,37].

**Figure 3:**
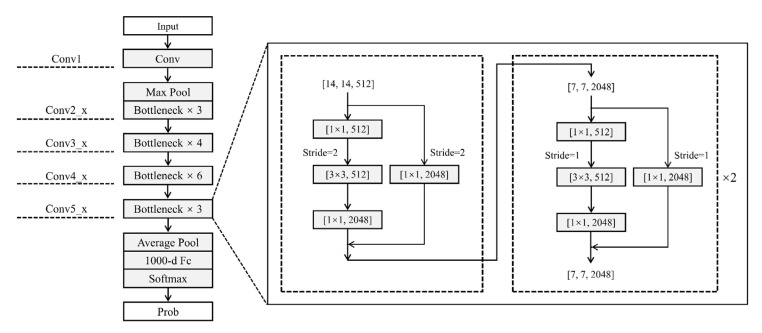
ResNet network structure.

The ResNet50 structure used in this study (Figure 3):

(1) The input data size is (224, 224, 11);
(2) After the first 7 × 7 convolutional layer, the output channel is 64 with a step size of 2 and a pad of 3;
(3) After the 3 × 3 maximum pooling layer, the step size is 2 and pad is 1;
(4) After conv2_x, this stage has the same fast superimposed residuals. Both input size and output size are 56 × 56;
(5) The first residual block of the conv3_x, conv4_x and conv5_x stacks are slightly different from the other residual blocks.

#### 2.3.2. ViT

**Figure 4:**
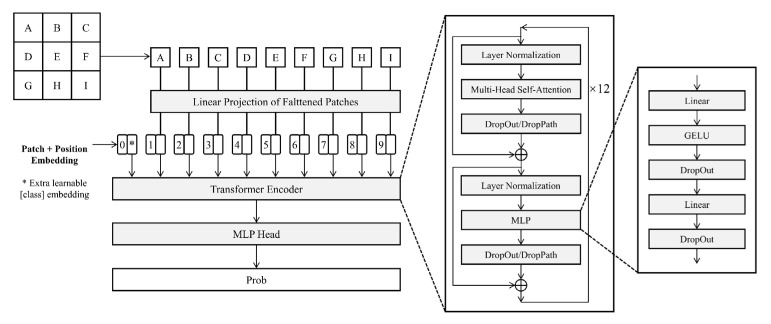
ViT network structure.

ViT is based on a Transformer encoder-based model in which the input image is chunked using Patch and Position Embedding. The segmented image blocks are combined into a sequence to obtain the sequence information. The sequence information is then passed to the Transformer Encoder for feature extraction, with the aim of adding a Classtoken to the image sequence. During the extraction process, the Classtoken interacts with other features, fusing features from other image sequences. After feature extraction, the Classtoken is fully concatenated for classification using MHSA (Figure 4). In this paper, the ViT-B/16 model, a derivative of ViT, is chosen, consisting of a stack of 12 blocks, each containing 16 attentional mechanisms [38,39].

#### 2.3.3. CNN and ViT Fusion

**Figure 5:**
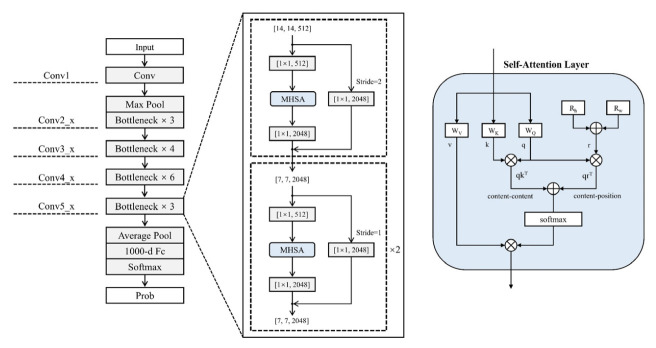
BoTNet network structure.

Most of the commonly used CNNs use 3 × 3 convolutional kernels. Convolutional operations can effectively extract local information, but for some vision tasks such as target detection, instance segmentation and key point detection, long-range dependencies need to be established. Self-Attention can effectively learn the association between each pair of entities and avoid stacking multiple convolutional layers in order to aggregate local information [40]. At the same time, the fusion of CNN and Attention can solve the fixed size problem of input data faced by current ViT. BoTNet incorporates the Attention module into the CNN block [41]. In this paper, the last three blocks of ResNet are replaced with BoTNet, and the rest are left unchanged. In other words, only the last three 3 × 3 convolutions of ResNet50 are replaced with MHSA layers (Figure 5). This approach significantly improves the baseline in terms of instance segmentation and target detection while also reducing the parameters, thereby minimising latency. This hybrid design can effectively exploit the advantages of convolution and Self-Attention, while downsampling through convolution allows for the efficient processing of higher-resolution input images [42].

**Figure 6:**
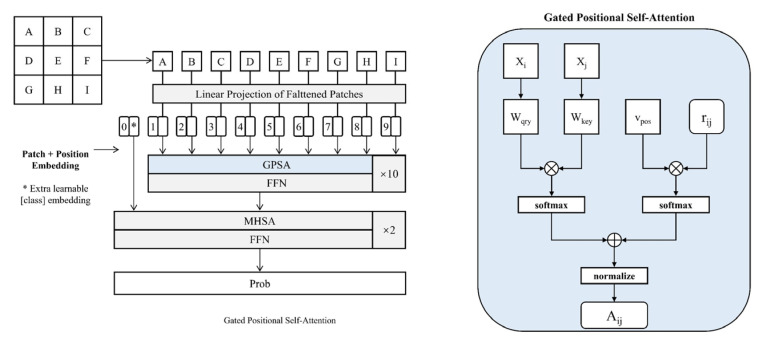
ConViT network structure.

ConViT combines two widely used AI architectures, CNN and Transformer. The model takes the strengths and weaknesses and overcomes some of the limitations of CNN and Transformer themselves (Figure 6). ConViT builds on ViT is adapted to take advantage of the soft convolutional induction bias in order to motivate the network to perform convolutional operations. At the same time, ConViT allows the model to decide for itself whether to maintain convolution or not. To exploit this soft induction bias, a form of positional Self-Attention called “Gated Positional Self-Attention (GPSA)” is used, where the model learns the gating parameter lambda, which is used to balance the content-based Self-Attention with the convolutional initialisation position. attention and convolutional initialised positional Self-Attention. In addition to the performance benefits of ConViT, the gating parameter provides a simple way to understand the degree of convolution at each layer after the model is trained. ConViT pays progressively less attention to convolutional positional attention during the training process. For the leaning layers, the gating parameter eventually converges to close to 0, indicating that the convolutional induction bias is effectively ignored. However, for the starting layers, many attention heads maintain high gating values, suggesting that the network uses the convolutional induction bias of the earlier layers to aid training [43].

## 3. Experiments and Results

**Figure 7:**
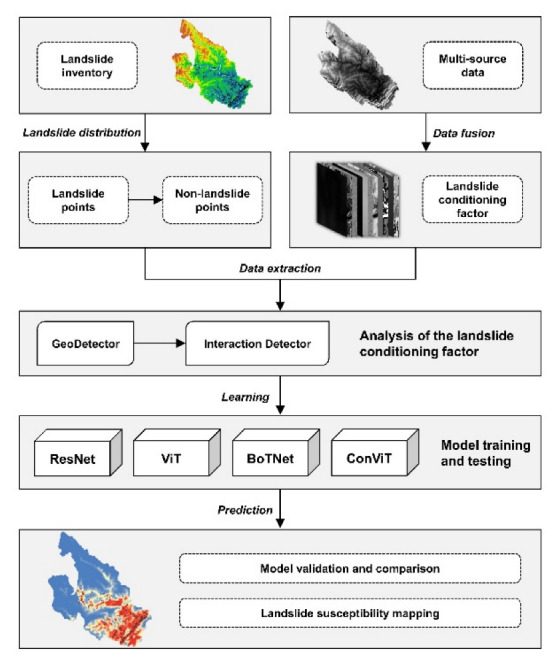
Flow chart of this study.

In this paper, firstly, we construct the landslide dataset and analyse whether there is a co-linearity problem between the landslide conditioning factors. Then, the landslide dataset is divided into a training dataset and a test dataset according to 70%:30%, which are used for training and evaluation of the models, and the best weights of each model are saved. Finally, the entire data from the study area, which was input into the saved models, was used to calculate the probability of landslide occurrence for the entire study area. The flow chart for this study is shown in Figure 7.

### 3.1. Constructing Landslide Datasets

We sampled an equal number of non-landslide points at 1 km intervals outside the 2 km buffer zone of the landslide site and within the study area to collectively form the landslide dataset. The landslide dataset is centred on 111 raster cells to the west and north, and 112 raster cells to the east and south, forming a single conditioning factor slice with a data dimension of 224 × 224. All 11 landslide conditioning factors are sliced in the same way as above to form the evaluation data for the landslide site with a dimension of 224 × 224 × 11. The landslide conditioning factors and the landslide dataset are normalised before being entered into the model.

The interaction detection of the geographical detectors is to identify whether the different conditioning factors *X*1 and *X*2 together increase or decrease the explanatory power of the dependent variable on *Y* or whether the effects of these factors on *Y* are independent of each other. This is assessed by first calculating the *q*-values of the two factors *X*1 and *X*2 on *Y* separately: *q(X*1*)* and *q(X*2*)* and calculating the *q*-values of their interactions [44].

(1) $$q=1-\frac{\sum_{h=1}^{L}N_{h}\sigma_{h}^{2}}{N\sigma^{2}}=1-\frac{SSW}{SST}$$

where *h =* 1, …, *L* is the classification of variable *Y* or factor *X*; *N_h_* and *N* are the number of cells in stratum *h* and the whole region, respectively, and $\sigma_{h}^{2}$ and $\sigma^{2}$ are the variances of *Y* values in the stratum *h* and the whole region, respectively. *SSW* is the sum of variances within the stratum, and *SST* is the sum of variances in the whole region. *q* has a value range of [0, 1], and larger *q* values indicate more pronounced spatial heterogeneity of *Y*; if the stratum is generated by the independent variable *X*, then a higher value of *q* indicates a stronger explanatory power of the independent variable *X* on the attribute *Y* and weaker reproduction. Table 2 shows that the *q*-value of the interactions of any two landslide conditioning factors on landslides is greater than the *q*-value of a single factor and greater than the sum of the *q*-values of the two factors. Therefore, there is no covariance between the 11 landslide conditioning factors chosen for this paper [45].

### 3.2. Model Evaluation

The landslide dataset was divided into a training dataset and a test dataset in a 70%:30% ratio and input to each classification model to extract the probability of its classification as a landslide. We used accuracy, *F*1-*score* and receiver operating characteristic (ROC) curves to evaluate the differences between the models. *TP* in Equations (2) and (3) is True Position, *TN* is True Negative, *FP* is False Position and *FN* is False Negative, and all of the above metrics can be solved by confusion matrices. The ROC curve of the model and the AUC (area under the curve) value are also calculated [46].

(2) $$accuracy=\frac{TP+TN}{TP+TN+FP+FN}$$

(3) $$F1-score=\frac{TP}{2\times TP+FP+FN}$$

As shown in Table 3, all four models showed high accuracy on the test dataset, with BoTNet and ConViT having the highest accuracy, again at 87.78%. The *F*1-*score* metric of ConViT was the highest at 87.64%, and the *F*1-*score* of the remaining three models were all greater than 85%. Table 3 also shows the evaluation metrics for the training dataset of each model. The four models did not show significant differences in the accuracy and *F*1-*score* on the training and test datasets, indicating that none of the four models involved in this paper showed overfitting.

**Figure 8:**
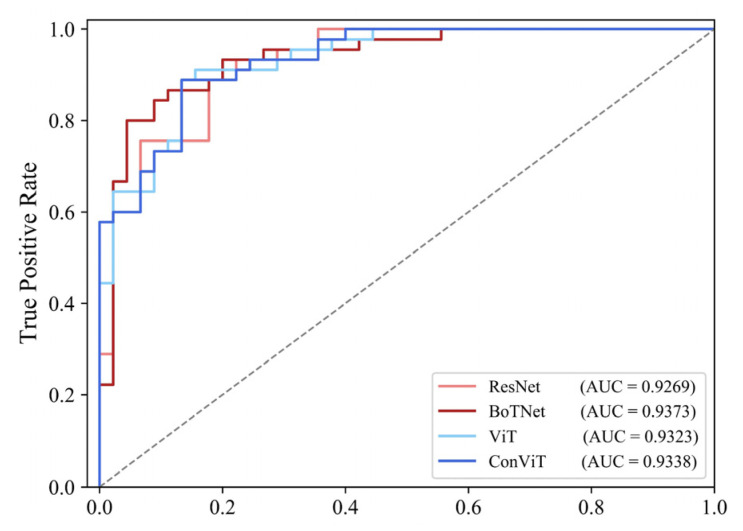
ROC curve of different LSM models based on the testing dataset.

Table 3 and Figure 8 show the ROC plots and AUC values for the four models. the AUC values for all four models are greater than 0.9, indicating that the method chosen in this paper is well suited to the study area. Table 4 shows the hyperparameter settings for each model.

### 3.3. Landslide Susceptibility Mapping

**Figure 9:**
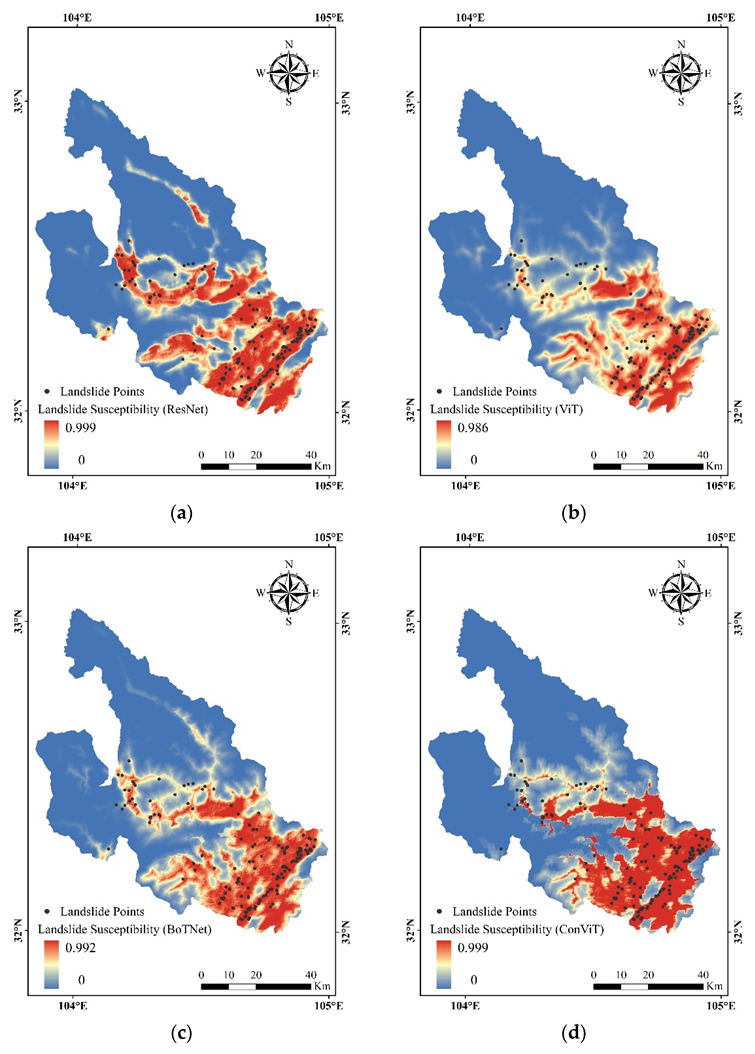
Landslide susceptibility mapping by (**a**) ResNet, (**b**) ViT, (**c**) BoTNet and (**d**) ConViT.

**Figure 10:**
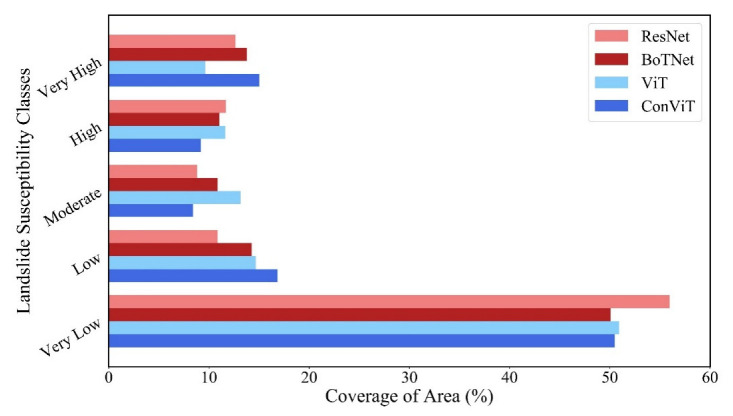
Landslide probability rating of the study area.

We input the full data of the study area into the trained models and the LSMs of the four models are shown in Figure 9. The results show that the area with a high probability of landslide occurrence is located in the southeast of the study area. We combined geospatial data and field surveys to find that this area has a relatively high concentration of historical landslide sites, with more pronounced elevation relief and closer proximity to rivers, and that this area contains faults. As a result of these factors, the south-eastern part of the study area shows a large area of high landslide susceptibility. Figure 10 shows the results of the models using natural interval statistics, which are divided into five classes: very low susceptibility, low susceptibility, moderate susceptibility, high susceptibility and very high susceptibility. Four models predicted the highest percentage of very low susceptibility zones, all of which exceeded 50%. The predictions of the four models showed some similarity in terms of spatial layout and zoning statistics, and were consistent with the actual situation, indicating that the results of the four models showed high reliability.

## 4. Discussion

### 4.1. Impact of MHSA in CNN

**Figure 11:**
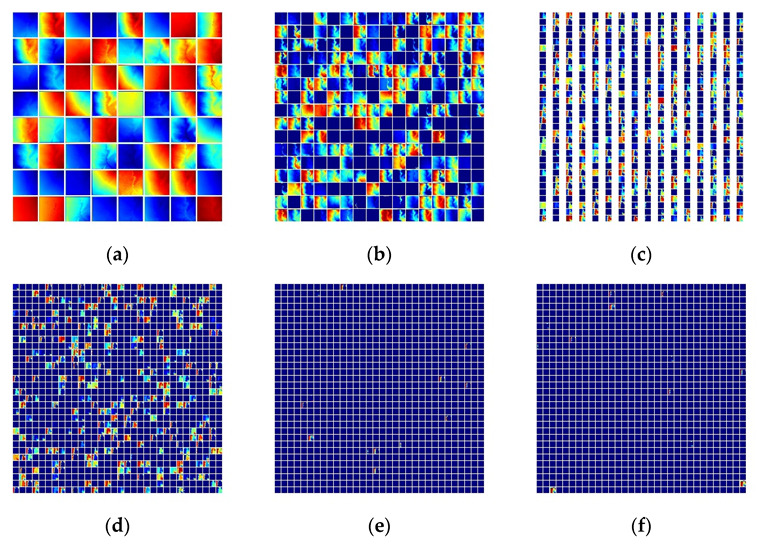
Feature map of ResNet; (**a**) conv1, (**b**) layer1, (**c**) layer2, (**d**) layer3, (**e**) layer4_1 and (**f**) layer4_2.

**Figure 12:**
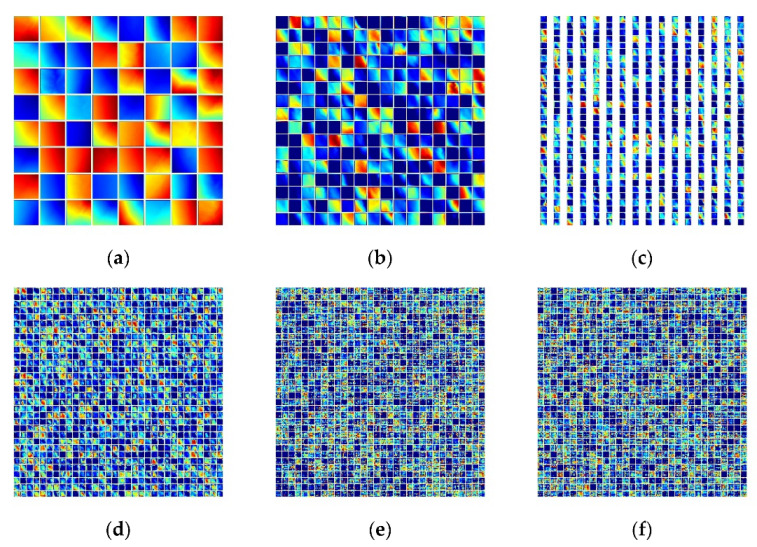
Feature map of BoTNet; (**a**) conv1, (**b**) layer1, (**c**) layer2, (**d**) layer3, (**e**) layer4_1 and (**f**) layer4_2.

BoTNet, compared to ResNet, only uses MHSA to replace the 3 × 3 convolutional layers in the conv5_x stage, reflecting part of the difference in terms of model accuracy and prediction results. We extracted the feature heat maps of conv1_x, layer1, layer2, layer3 and layer4 during prediction for ResNet and BoTNet, respectively, as shown in Figure 11 and Figure 12. The two CNN-based models reflect a huge difference in the amount of effective information in layer3 and layer4.

**Figure 13:**
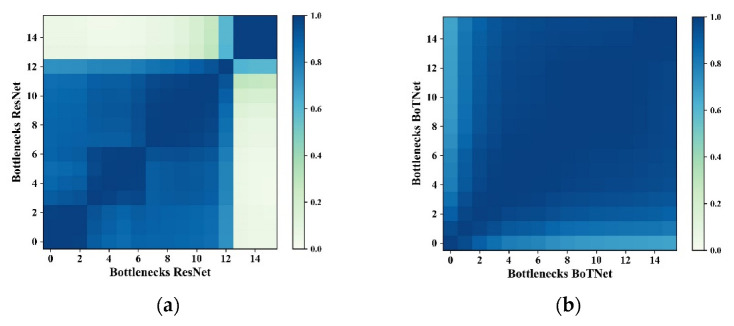
Feature map similarity. (**a**) ResNet and (**b**) BoTNet.

Meanwhile, we used the Centered Kernel Alignment (CKA) [47] method to calculate the similarity between each Bottleneck of ResNet and BoTNet so as to achieve a quantitative comparison of feature similarity within the model. The *x*-axis and *y*-axis indicate the index of Bottleneck. Figure 13 shows that there is a high similarity between Bottleneck within each of the ResNet conv, a relatively low similarity between Bottleneck between different conv_x, and a low similarity between the deep and shallow layers. The BoTNet model shows high similarity [47]. In BoTNet, we can see that the overall colour indicates that similar representations are obtained, regardless of the depth of the layers. In ResNet, on the other hand, we notice no similarity between the representations obtained in the shallow and deep layers. This could be because, in BoTNet, we get the global representation from the beginning, whereas, in ResNet, we need to propagate the layers to get the global representation.

### 4.2. Model Adaptation and Stability

**Figure 14:**
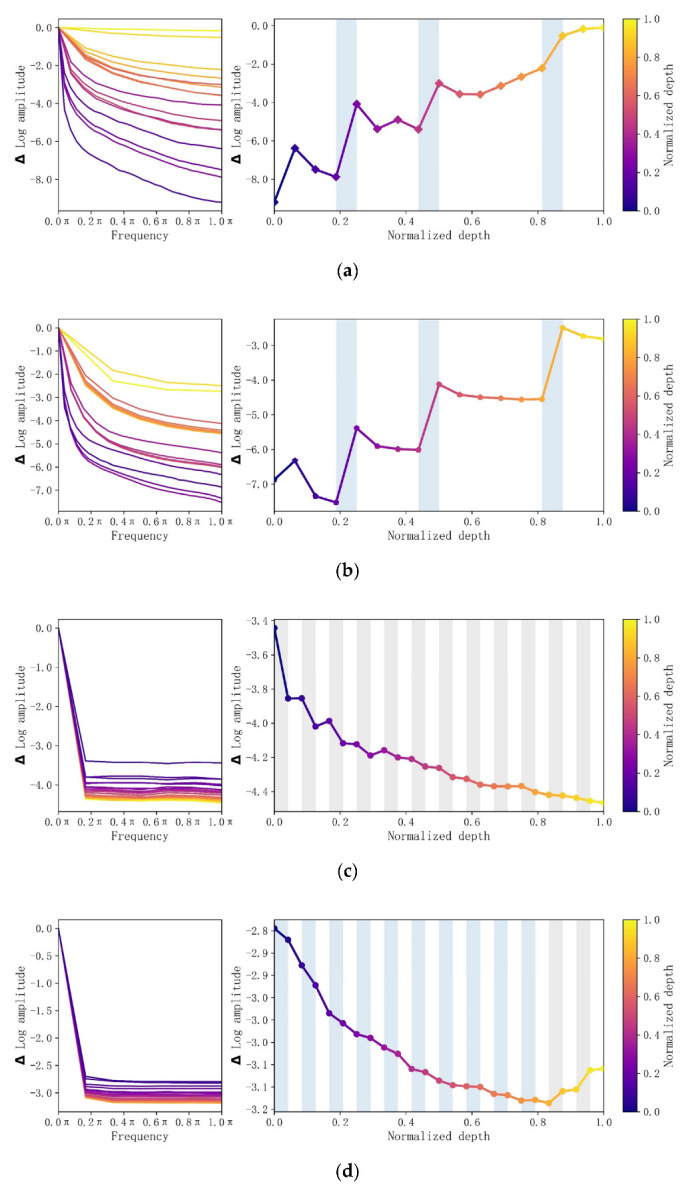
Relative log amplitude of the Fourier transform of the model feature maps (**a**) ResNet, (**b**) BoTNet, (**c**) ViT and (**d**) ConViT.

To verify the best working scenario for the model, we calculated the relative log amplitude of the Fourier transform of the four model feature maps [29]. As shown on the left-hand side of Figure 14, the Δlog amplitude of the high-frequency signal is the difference in log amplitude at the normalised frequencies of 0.0*π* (centre) and 1.0*π* (boundary). The right-hand side of Figure 14 shows the relative log amplitude of each layer, with the white, grey and blue areas indicating the conv/MLP, MHSA and downsample/subsample layers, respectively. The MHSA in ViT and the GPSA in ConViT tend to reduce high-frequency noise, while each conv_x phase of ResNet and BoTNet, in contrast, is increasing high-frequency noise. Since low-frequency signals tend to affect the performance of ViT and high-frequency signals tend to affect the performance of ResNet, while low-frequency signals correspond to the shape of the image and high-frequency signals correspond to the texture of the image [48,49,50]. Therefore, we believe that the ViT model will pay more attention to the shape of the landslide unit and the ResNet model will pay more attention to the texture of the landslide unit [29].

**Figure 15:**
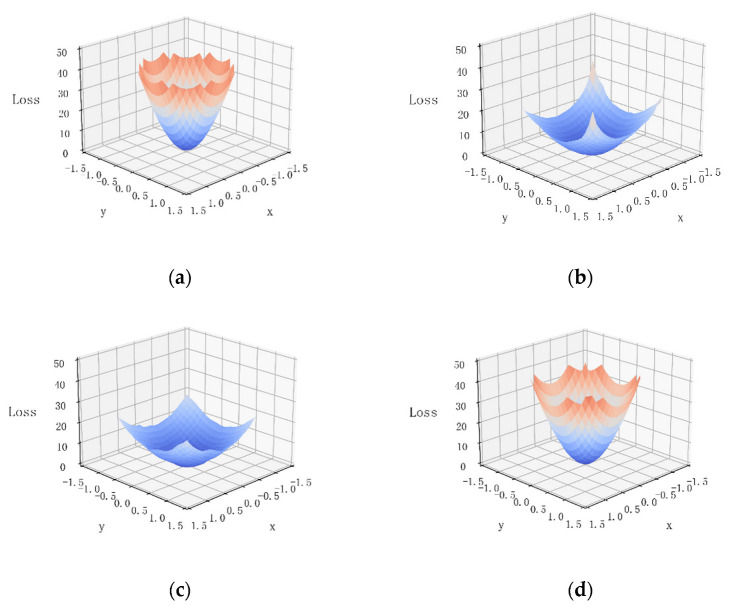
Loss landscapes. (**a**) ResNet, (**b**) BoTNet, (**c**) ViT and (**d**) ConViT.

We have visualised the loss functions for each model (Figure 15). The loss in the figure is the Hessian matrix eigenvalue of the loss function (the loss function is augmented with L2 regularisation). Figure 15 shows that the loss landscapes are more similar for ViT and BoTNet, and the loss landscapes results are more similar for ResNet and ConViT. The inclusion of the MHSA module in ViT and BoTNet makes the loss landscapes of the models smoother, which in turn makes model optimisation more difficult [29]. On the other hand, CNN’s loss landscapes are more prominent and steeper, and are more likely to fall into local optima than ViT, so ViT’s loss landscapes are not at a disadvantage relative to CNN. The flat loss landscapes also mean that the model is more generalisable, and the model is more malleable. With the addition of some CNN elements to ConViT and MHSA elements to BoTNet, the computational results of both fusion models converge in the direction of the newly added elements [29].

### 4.3. Existing Problems and Future Research

The study area chosen for this paper is located at the confluence of the Tibetan Plateau and the Sichuan Basin, where the topography is highly undulating. The selected study area has some specificity and future experiments should be conducted in several study areas. The accuracy of some of the raw data is 30 m spatial resolution, which is different from the accuracy of the topographic data, and this may reduce the accuracy of the predictions.

## 5. Conclusions

This paper applies the fused two models of CNN (ResNet) and ViT to the LSM domain. Using Pingwu County, Sichuan Province, China, as the study area, 11 landslide conditioning factors are selected to predict the probability of landslides occurring in the study area based on the historical landslide dataset in the study area. The classification accuracy and generalisation ability of BoTNet and ConViT models are also analysed, and finally the results of LSM are synthesised with the actual situation in the study area to draw the following conclusions.

(1) The single classification models involved in this study, ResNet and ViT, both exhibit better classification accuracy and generalisation ability. Among them, ViT has higher accuracy, and the prediction results are more in line with the actual situation.
(2) The fusion model of CNN and ViT, which was applied to the field of landslide susceptibility mapping for the first time, showed better applicability. The fused model outperformed the single classification model in terms of performance.
(3) The fusion model of CNN and ViT can effectively suppress high-frequency noise and take into account the texture and shape of landslide units at the same time.

The two fused models chosen in this paper do not fuse the CNN with ViT in a deeper way. In a subsequent study, we can try to fuse CNN with Vision Transformer in a deeper way, using CNN in the first half of the model and ViT in the second half. The advantages of doing so are twofold: on the one hand, it can effectively suppress the high-frequency noise in the model, and on the other hand, it can better take into account the shape and texture of the landslide units [29] and more fully exploit the advantages of convolution and MHSA in different scenes.

## Figures and Tables

**Table 1:** Summary of data.

Data Layer	Source	Scale/Resolution	Data Type	Data Summary			
				5th Percentile	95th Percentile	Mean	Standard Deviation
Elevation	China Academy of Surveying and Mapping Science Beijing Four Dimensions Digital Technology Co	10 m	Continuous	944.49	3669.44	2905.84	861.38
Slope		10 m	Continuous	11.72	54.23	33.43	12.94
Aspect		10 m	Continuous	18.72	341.42	178.67	101.51
Plan curvature		10 m	Continuous	−4.94	4.98	0.02	3.02
Profile curvature		10 m	Continuous	−8.54	8.57	0.02	5.14
Distance to river		10 m	Continuous	500.00	13,222.90	5908.10	4030.09
Distance to fault		10 m	Continuous	644.05	17,353.53	8398.08	5273.34
Lithology	Chinese Academy of Science	10 m	Categorical	-	-	-	-
Rainfall		30 m	Continuous	7375.00	8463.00	7918.62	324.63
Landcover		30 m	Categorical	-	-	-	-
NDVI		30 m	Continuous	107.00	207.00	176.37	31.44

**Table 2:** The *q*-value of the interactions *.

	Elevation	Slope	Aspect	Plan Curvature	Profile Curvature	Rainfall	NDVI	Lithology	Landcover	Distance of River	Distance of Fault
Elevation	-										
Slope	B	-									
Aspect	N	N	-								
Plan Curvature	N	N	N	-							
Profile Curvature	N	N	N	N	-						
Rainfall	B	B	N	N	N	-					
NDVI	B	N	N	N	N	N	-				
Lithology	B	B	N	N	N	N	N	-			
Landcover	B	B	B	N	N	B	B	B	-		
Distance of River	B	N	N	N	N	B	N	B	B	-	
Distance of Fault	N	N	N	N	N	N	N	N	N	N	-

* N for nonlinear enhancement, and B for bilinear enhancement.

**Table 3:** Evaluation result of LSM models.

Method	Train Dataset			Test Dataset		
	Accuracy (%)	*F*1-*Score* (%)	AUC (%)	Accuracy (%)	*F*1-*Score* (%)	AUC (%)
ResNet	86.67	88.28	96.07	86.00	85.71	92.69
ViT	89.05	88.78	96.99	86.67	86.36	93.23
BoTNet	90.00	89.76	97.52	87.78	87.36	93.73
ConViT	88.10	88.04	94.18	87.78	87.64	93.38

**Table 4:** Hyperparameter settings of models for LSM.

Method	Hyperparameter					
	Epoch	Batch Size	Dropout	Optimiser	Loss Function	Learn Rate
ResNet	300	16	0	Adam	CrossEntropyLoss	0.01
ViT	300	16	0	SGD	CrossEntropyLoss	0.001
BoTNet	300	16	0.3	SGD	CrossEntropyLoss	0.0001
ConViT	300	16	0	Adam	CrossEntropyLoss	0.0001
